# Risk prediction of sleep disturbance in clinical nurses: a nomogram and artificial neural network model

**DOI:** 10.1186/s12912-023-01462-y

**Published:** 2023-08-28

**Authors:** Xinyu Zhang, Lei Zhang

**Affiliations:** 1 The First Affiliated Hospital of Jinzhou Medical University, 121001 Jinzhou, People’s Republic of China; 2 Department of Nursing, Jinzhou Medical University, No.40, Section 3, Songpo Road, Linghe District, 121001 Jinzhou, People’s Republic of China

**Keywords:** Clinical nurse, Sleep disturbance, Nomogram, Artificial neural network, Prediction model

## Abstract

**Background:**

Sleep disturbance occur among nurses at a high incidence.

**Aim:**

To develop a Nomogram and a Artificial Neural Network (ANN) model to predict sleep disturbance in clinical nurses.

**Methods:**

A total of 434 clinical nurses participated in the questionnaire, a cross-sectional study conducted from August 2021 to June 2022.They were randomly distributed in a 7:3 ratio between training and validation cohorts.Nomogram and ANN model were developed using predictors of sleep disturbance identified by univariate and multivariate analyses in the training cohort; The 1000 bootstrap resampling and receiver operating characteristic curve (ROC) were used to evaluate the predictive accuracy in the training and validation cohorts.

**Results:**

Sleep disturbance was found in 180 of 304 nurses(59.2%) in the training cohort and 80 of 130 nurses (61.5%) in the validation cohort.Age, chronic diseases, anxiety, depression, burnout, and fatigue were identified as risk factors for sleep disturbance. The calibration curves of the two models are well-fitted. The sensitivity and specificity (95% CI) of the models were calculated, resulting in sensitivity of 83.9%(77.5–88.8%)and 88.8% (79.2–94.4%) and specificity of83.1% (75.0–89.0%) and 74.0% (59.4–84.9%) for the training and validation cohorts, respectively.

**Conclusions:**

The sleep disturbance risk prediction models constructed in this study have good consistency and prediction efficiency, and can effectively predict the occurrence of sleep disturbance in clinical nurses.

## Introduction

Sleep is a normal physiological phenomenon, which is spent about 1 / 3 of a person’s life [1] Related studies have shown that sleep has the functions of saving and storing body energy, regulating body heat and metabolism, promoting cell tissue repair after injury, enhancing brain cognition, delaying memory decline and so on, which is closely related to our physical and mental health [2] , 3] However, Sleep disturbance is common among clinical nurses.The incidence of sleep disturbance among domestic nurses has reached 55.38% [4] ]. Sleep disturbance can lead to sleepiness, fatigue, lethargy, and other serious work-related consequences, such as work errors [5] .

Due to the particularity of the nursing profession, the nature of high-intensity and high-stress work, 6] the night shifts 7] and the continuous learning of advanced medical knowledge and technology, the various needs of nursing work do not adapt to their physical and mental state, resulting in job burnout, 8] affecting the quality of sleep. At the same time, nurses’ working environment, work intensity, interpersonal relationship with patients and colleagues, and social role disturbance also lead to higher stress than other professional groups, Thus, nurses become a high group of sleep disturbance.And their sleep problems are more easily overlooked [9] Good sleep quality is the key for nurses to provide the best care for patients. Therefore, it is very important for nurses in the clinical to screen for the risk of sleep disturbance and early intervention. However, at present, the research on nurses’ sleep problems in China is limited to the investigation of the current situation, A reliable tool for identifying the risk of nurses’ sleep disturbance has not been established. Practical and reliable predictive models are needed to predict the occurrence of sleep disturbance. Nomograms are statistical models specifically designed to maximize predictive accuracy, which can allow for an individualized prediction of outcomes [10] ANN (artificial neural network) as a computer technology has also been shown to outperform traditional discriminant analysis by determining the importance of each variable for a particular event and predicting outcomes based on the variables present [11] , 12] However, to our knowledge few studies have focused on using the above models to predict the risk of sleep disturbance.

Therefore, we conducted a cross-sectional study to develop a nomogram and ANN model valid for predict sleep disturbance in clinical nurses.

## Subjects and methods

### Subjects

This cross-sectional study was conducted between August 2021 and June 2022, and 444 male and female clinical nurses were approached.Nurses were selected based on convenience sampling.This study was approved by the ethics committee of the Jin zhou Medical University of traditional Chinese and followed the guidelines of the Declaration of Helsinki. Written consent was obtained from all participants. Ultimately, complete responses were obtained from 434 nurses, for a response rate of 97.7%. They were randomly allocated to either the training cohort or the validation cohort at a ratio of 7:3.The inclusion criteria were: (a) registered nurse with qualification certificate; (b) with at least 1 year of experience.The exclusion criteria were:(a) retired nurses; or (b) trainee nurses. (c)nurses with severe sleep disturbances,severe stressful events, or physical or mental illness.

### Instruments

#### General demographic characteristics questionnaire

This form was used to gather information on the participant’s gender,age, education level, marital status,fertility status, chronic illness, way of appointment,night shifts and tea or coffee usage during work.

#### The pittsburgh sleep quality index (PSQI)

The PSQI, 13] was designed to assessed sleep quality. The PSQI consists of seven components: sleep latency, sleep duration, habitual sleep efficiency, sleep disturbances, daytime dysfunction, and hypnotics, each section score ranges from 0 to 3 points.PSQI total scores range from 0 to 21 and are obtained by summing the scores of the seven components. A total score of 7 or higher was considered to have a sleep disturbance.(Cronbach’s α = 0.73).

#### The maslach burnout inventory (MBI)

The MBI, 14] was designed to measure burnout. The questionnaire consists of 22 items in descriptive form related to personal feelings and attitudes and is composed of three subscales. The Emotional Exhaustion subscale (EE) (9 items), the Depersonalization subscale (DP) (5 items), and the Personality Coping subscale (PA) (8 items). The scoring criteria were as follows: EE subscale scored 27 or above, DP subscale scored 10 or more, PA subscale scored 33 or below was considered “high Burnout”; EE subscale scored 19–26, DP subscale scored 6–9, PA subscale scored 34–39 was considered “moderate Burnout”; EE subscale scored18 or below, DP subscale scored 5 or below and PA subscale scored 40 or below were considered “low Burnout”.(Cronbach’s α = 0.84).

### Fatigue scale-14(FS-14)

The FS-14, 15] was designed to measure the severity of fatigue symptoms. The scale consists of 14 items that reflect different perspectives on the degree of fatigue, with items 1–8 reflecting physical fatigue and items 9–14 reflecting mental fatigue; scores from items 1–8 are summed to form the physical fatigue score, scores from items 9–14 are summed to form the mental fatigue score, the sum of the physical fatigue score and mental fatigue score is the total fatigue score. The highest physical fatigue score is 8, the highest mental fatigue score is 6, and the total scale score is 14. A total score of 7 or higher indicates a state of fatigue.(Cronbach’s α = 0.81).

#### Hospital anxiety and depression scale(HADS)

The HADS, 16] was used to determine anxiety and depression symptoms.The scale consists of 14 items: 7 items assessing anxiety and 7 items assessing depression. The scoring criteria are as follows: scale score 0–7: no depression or anxiety disturbance; scale score 8–10: mild depression or anxiety disturbance; scale score 11–14: moderate disturbance; scale score 15–21: severe disturbance.(Cronbach’s α = 0.88).

### Data collection

Before the study began, each participant was informed of the purpose of the study and of his or her right to refuse or discontinue participation in the study at any time, while maintaining the anonymity and confidentiality of all participants. The questionnaires were distributed by a member of the research team. Before distributing the questionnaires, the purpose and importance of the study were fully explained and instructions were given on how to complete the questionnaires so that there would be no ambiguity in the answers to the questions. The questionnaires were answered anonymously, so participants’ privacy was not violated. The questionnaires were collected by the research team immediately after completion. If more than 10% of the questionnaire items were unanswered, they were considered invalid.

### Statistical analysis

Categorical variables are described as frequencies and percentages,statistical analyses were performed using SPSS 25.0 software for Windows (IBM, Somers, NY). In the training cohort, possible predictors of sleep disturbance were analyzed by univariate logistic regression analysis and variables with p < 0.05 in univariate analysis were considered candidate variables for multivariate logistic regression analysis. p < 0.05 was considered a sign of statistical significance in multivariate analysis. Nomogram and ANN models were constructed based on the results of multivariate logistic regression analysis of the training cohort. Nomogram was constructed using the strategic regression modeling package in R (version 4.0.2; R Project for Statistical Computing; www.rproject.org). After building the predictive model, the accuracy and discriminative power of the internal (training cohort) and external (validation cohort) models were tested using 1000 bootstrap resampling and ROC curves (receiver operating characteristic).

## Results

### Participant characteristics

**Fig. 1:**
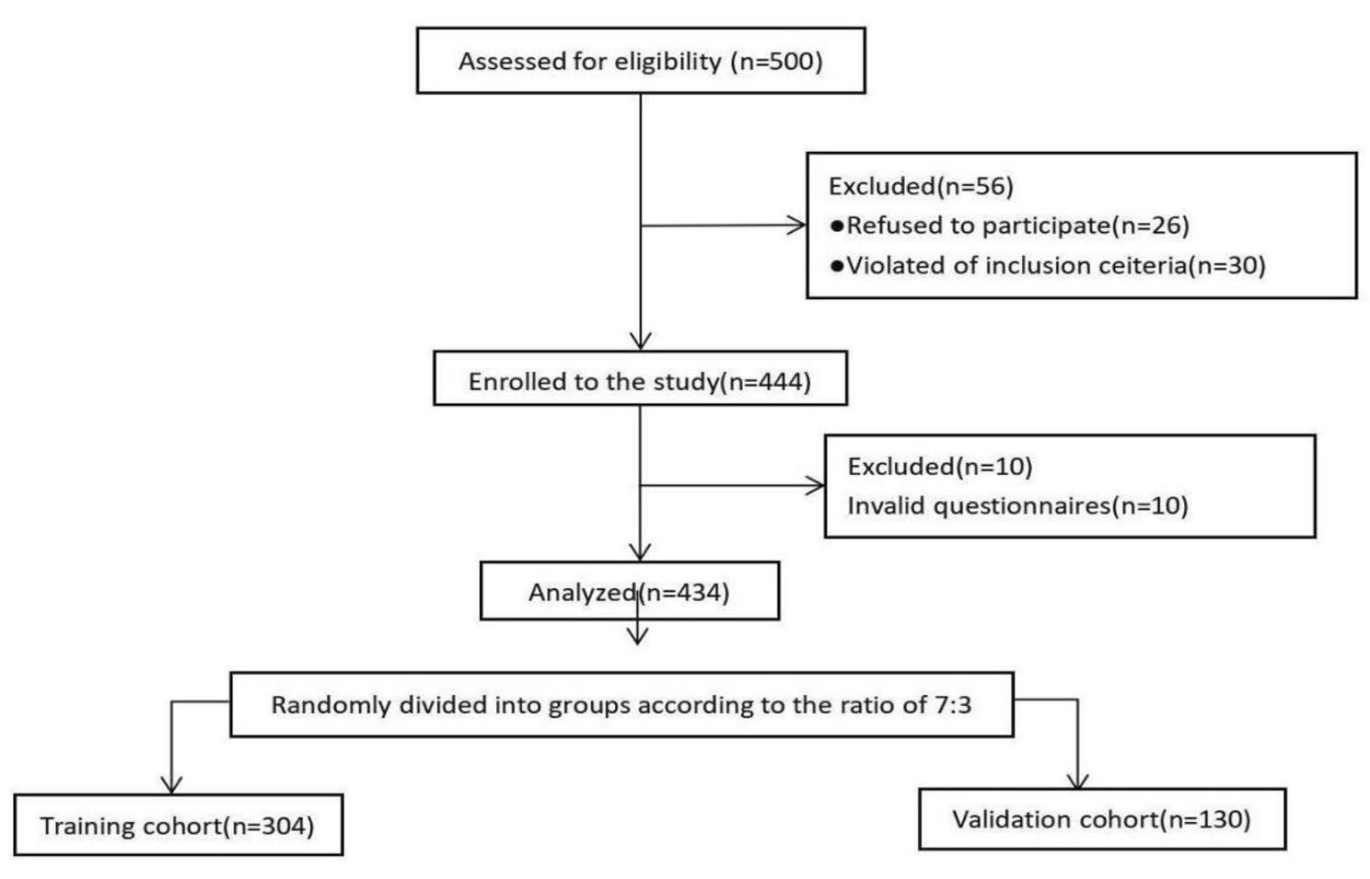
Flow chart of patients screening and recruitment

We first assessed the eligibility of 500 clinical nurses to participate in our study (Fig. 1). Of these, 26 nurses refused to participate, 30 nurses did not meet the inclusion criteria, and the remaining 444 nurses participated in the study. After the study was completed, 10 invalid questionnaires were excluded from the study. Finally, the data of 434 nurses were analyzed in the present study. The training cohort composed of 304 nurses. 49.7% were age < 30years,14.8% were male and 59.2% had sleep disturbance. The validation cohort consisted of 130 nurses. 53.1% were age < 30years,17.7% were male and 61.5% had sleep disturbance.The prevalence of sleep disturbance among all nurses was 59.9%. All characteristics were no statistically significant differences between training cohort and validation cohort (all P > 0.05) (Table 1).

### Predictors of sleep disturbances in the training cohort

The results of univariable and multivariable logistic regression analyses for sleep disturbance are presented in Tables 2 and 3,respectively. On multivariate analysis, with results reported as odds ratio (95% CI), fatigue [2.76(1.40–5.45)], anxiety [3.44(1.60–7.41)], depression[3.73(1.83–7.59)], chronic diseases[4.05(1.91–8.59)], burnout [4.41(1.90-10.24)], age [7.33(1.56–34.41)], were independently associated with sleep disturbance.

**Table 1:** Participant Characteristics

Variables	Total(n = 434)	Training (n = 304)	Validation(n = 130)	χ 2]	P-Value
Sex				0.575	0.448
Male Female	68(15.7) 366(84.3)	45(14.8) 259(85.2)	23(17.7) 107(82.3)		
Age [years]				0.429	0.807
<30 30–40 >40	220(50.7) 150(34.6) 64(14.7)	151(49.7) 107(35.2) 46(15.1)	69(53.1) 43(33.1) 18(13.8)		
Education level				0.855	0.652
College degree and below Undergraduate Master degree and above	82(18.9) 308(71.0) 44(10.1)	54(17.8) 219(72) 31(10.2)	28(21.5) 89(68.5) 13(10.0)		
Marital status				0.478	0.489
Single Married	218(50.2) 216(49.8)	156(51.3) 148(4.78)	62(47.7) 68(52.3)		
Fertility				0.127	0.938
Not giving birth pregnancy Have given birth	290(66.8) 50(11.5) 94(21.7)	203(66.8) 36(11.8) 65(21.4)	87(66.9) 14(10.8) 29(22.3)		
Chronic diseases				0.001	0.981
None Yes	240(55.3) 194(44.7)	168(55.3) 136(44.7)	72(55.4) 58(44.6)		
Way of appointment Formally editing Contract system other	98(22.6) 256(59.0) 80(18.4)	64(21.1) 187(61.5) 53(17.4)	34(26.2) 69(53.1) 27(20.8)	2.698	0.260
Drink tea or coffe at work				0.395	0.529
NO Yes	154(35.5) 280(64.5)	105(34.5) 199(65.5)	49(37.7) 81(62.3)		
Shift work				2.096	0.148
NO Yes	210(48.4) 224(51.6)	154(50.7) 150(49.3)	56(43.1) 74(56.9)		
Fatigue				0.071	0.790
None Yes	161(37.1) 273(62.9)	114(37.5) 190(62.5)	47(36.2) 83(63.8)		
Anxiety				0.004	0.947
None Yes	246(56.7) 188(43.3)	172(56.6) 132(43.4)	74(56.9) 56(43.1)		
Depression				0.074	0.786
None Yes	228(52.5) 206(47.5)	161(53.0) 143(47.0)	67(51.5) 63(48.5)		
Burnout				0.140	0.932
Low Mederate High	132(30.4) 156(35.9) 146(33.6)	94(30.9) 109(35.9) 101(33.2)	38(29.2) 47(36.2) 45(34.6)		

**Table 2:** Risk Factors for Sleep Disturbance According to Univariate Analysis

Variable	Sleep disturbance			
	Normal sleep (n = 124)	Sleep disturbance (n = 180)	χ^2^/Z	P-value
Sex			1.435^a^	0.231
Male Female	22(48.9%) 102(39.4%)	23(51.1%) 157(60.6%)		
Age [years]			36.165^a^	0.000
<30 30–40 >40	83(55.0%) 38(35.5%) 3(6.5%)	68(45.0%) 69(64.5%) 43(93.5%)		
Education level			3.780^a^	0.151
College degree and below Undergraduate Master degree and above	16(29.6%) 93(42.5%) 15(48.4%)	38(70.4%) 126(57.5%) 16(51.6%)		
Marital status			27.278^a^	0.000
Single Married	86(55.1%) 38(25.7%)	70(44.9%) 110(74.3%)		
Fertility Not giving birth pregnancy Have given birth	106(52.2%) 8(22.2%) 10(15.4%)	97(47.8%) 28(77.8%) 55(84.6%)	33.484^a^	0.000
Chronic diseases			65.472^a^	0.000
None Yes	103(61.3%) 21(15.4%)	65(38.7%) 115(84.6%)		
Way of appointment			2.168^a^	0.338
Formally editing Contract system other	27(42.2%) 71(38.0%) 26(49.1%)	37(57.8%) 116(62.0%) 27(50.9%)		
Drink tea or coffe at			1.611^a^	0.204
work NO Yes	48(45.7%) 76(38.2%)	57(54.3%) 123(61.8%)		
Shift work			14.272^a^	0.000
NO Yes	79(51.3%) 45(30.0%)	75(48.7%) 105(70.0%)		
Fatigue			72.251^a^	0.000
None Yes	68(62.2%) 17(12.9%)	65(37.8%) 115(87.1%)		
Anxiety			57.141^a^	0.000
None Yes	107(60.9%) 26(18.2%)	63(39.1%) 117(81.8%)		
Depression			57.141^a^	0.000
None Yes	98(60.9%) 26(18.2%)	63(39.1%) 117(81.8%)		
Burnout			−4.604^b^	0.000
Low Mederate High	50(53.2%) 49(45.0%) 25(24.8%)	44(46.8%) 60(55.0%) 76(75.2%)		

**Notes**: Data are number (%). ^a^Chi-square test. ^b^Mann-Whitney

**Table 3:** Logistic Regression for the Predictors of Sleep Disturbance

Variable	Β Coefficient	OR(95%CI)	P-Value
Age(<30/30–40/≥40)	1.992	7.33(1.56–34.41)	0.012
Chronic diseases(Y/N)	1.400	4.05(1.91–8.59)	<0.001
Anxiety(Y/N)	1.235	3.44(1.60–7.41)	0.002
Depression(Y/N)	1.315	3.73(1.83–7.59)	<0.001
Burnout(Y/N)	1.485	4.41(1.90−10.24)	0.001
Fatigue(Y/N)	1.016	2.76(1.40–5.45)	0.003

### Development and validation of the nomogram and ANN

**Fig. 2:**
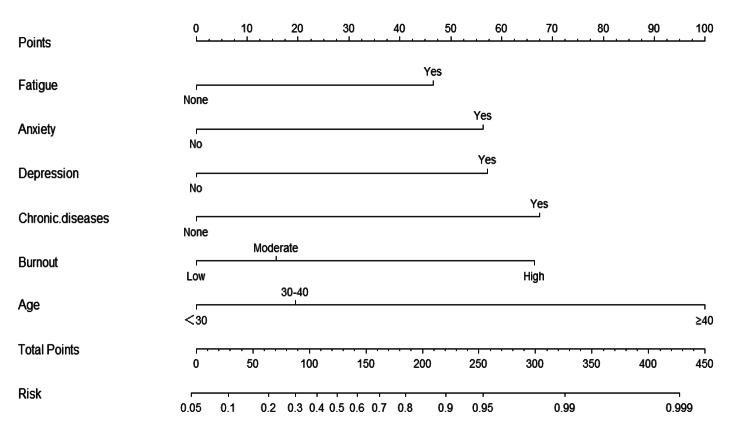
Sleep disturbance risk nomogram

**Fig. 3:**
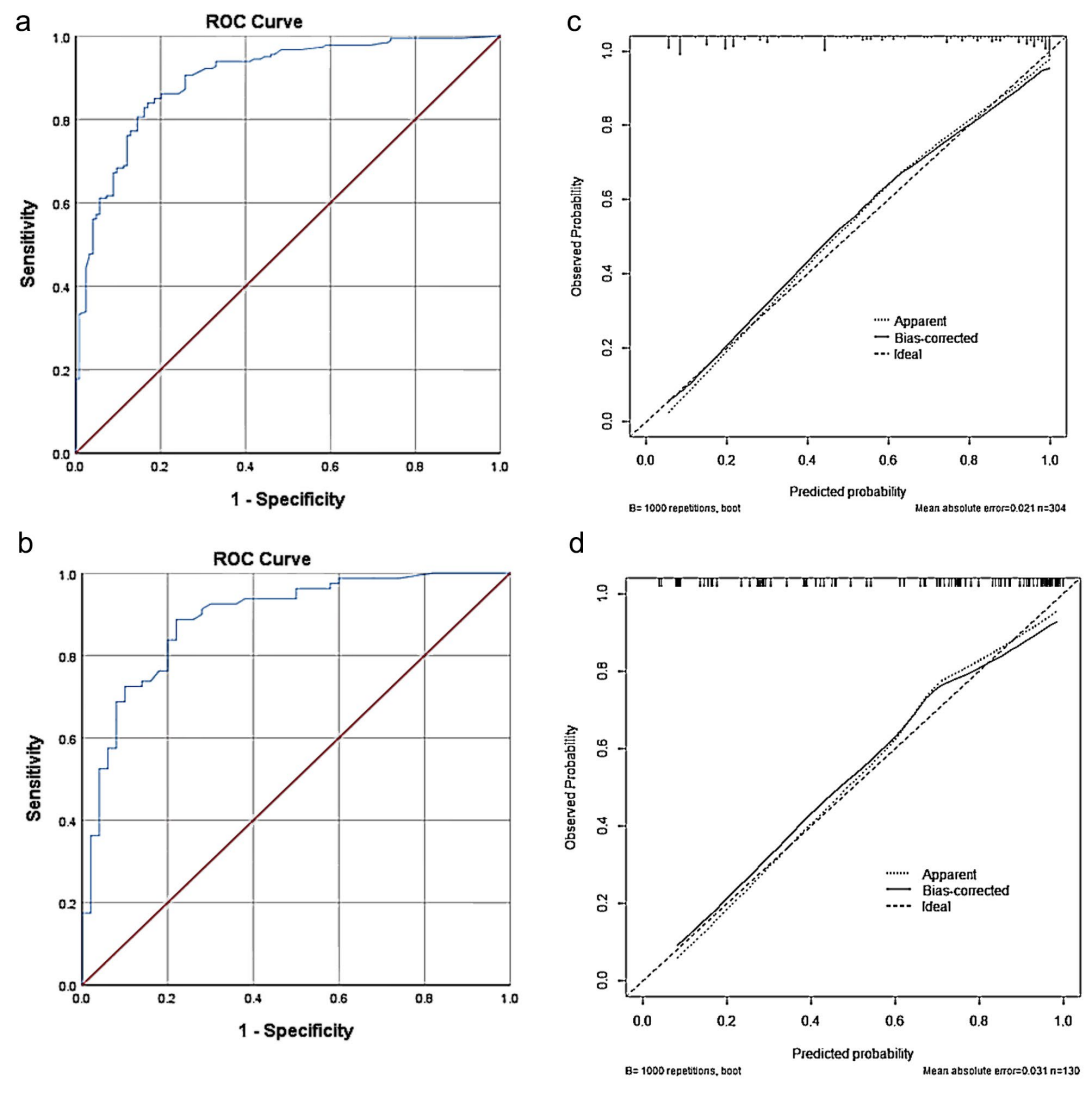
**a** The receiver operating characteristic (ROC) curve of training cohort. **b**:The receiver operating characteristic (ROC) curve of validation cohort. **c**:Validity of the predictive performance of the nomogram in estimating the risk of sleep disturbance presence in the training cohort (n = 304). **d**:Validity of the predictive performance of the nomogram in estimating the risk of sleep disturbance presence in the validation cohort (n = 130)

**Fig. 4:**
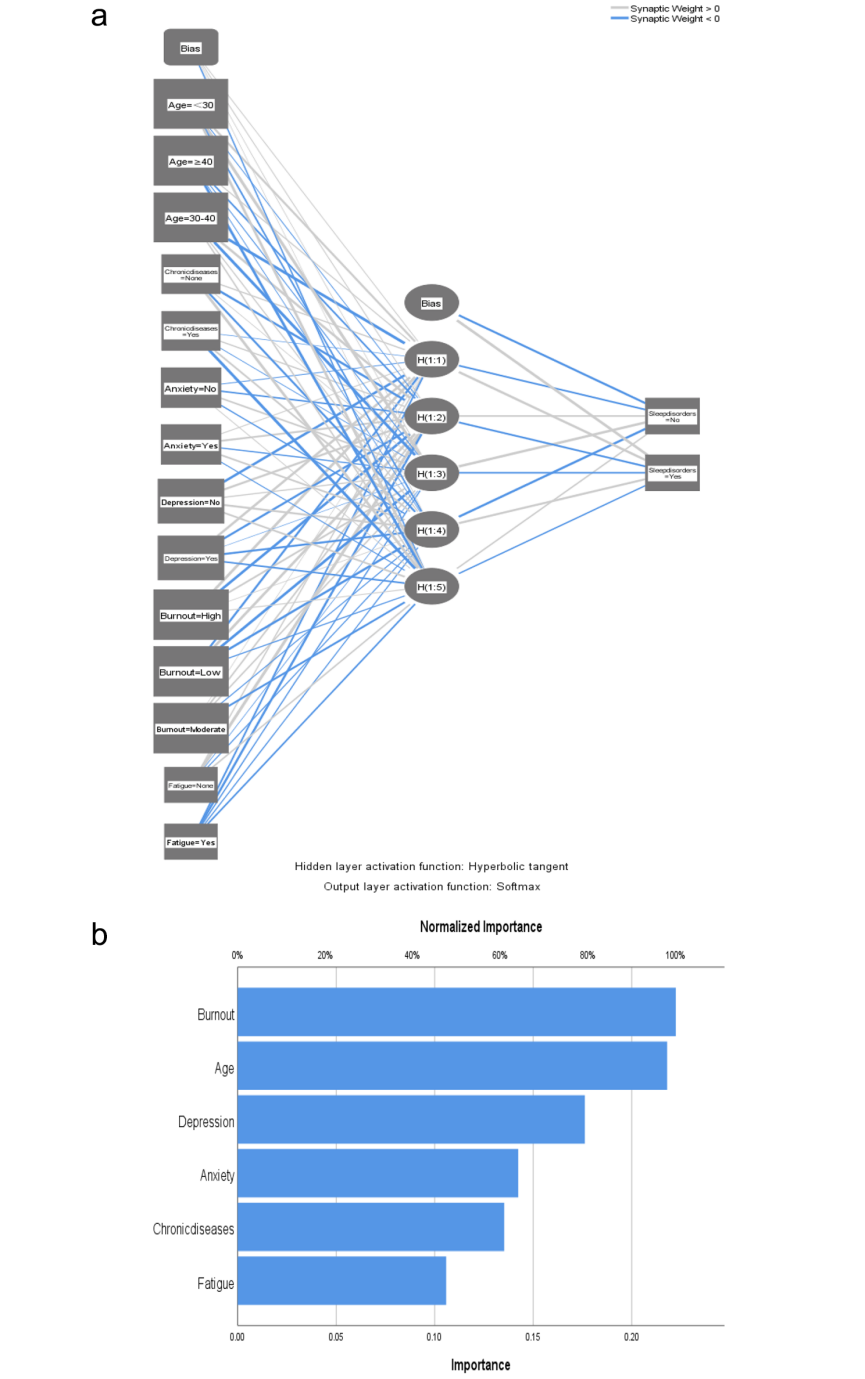
**a** Sleep disturbance risk ANN model. **b**: The importance of each variable in the artificial neural network (ANN) model

These independently associated risk factors were used to build a sleep disturbance risk prediction model (Fig. 2). Using the nomogram to find the position of each variable on the corresponding axis, draw a line on the Points axis, we could get the corresponding score of this risk factor. The total score was obtained by adding the scores of each factor. Projecting downward from the total score, the corresponding sleep disturbance risk prediction probability value could be obtained.The resulting model was internally validated using the receiver operating characteristic curve (ROC) and bootstrap validation method.According to the internal validation in the training cohort, the C-statistic of this nomogram was 0.90 (95% CI, 0.86–0.93); Fig. 3(a)),while the index of external validation in the validation cohort was 0.89(95% CI, 0.83–0.95); Fig. 3(b)).Besides, in the training and validation cohorts ,the calibration plots graphically demonstrated good agreement on the presence of sleep disturbance between the risk estimation by the nomogram and analysis results of actual clinical data (Fig. 3(c),3(d)). An ANN model was established based on the results of univariate analyses and multivariate logistic regression analyses. The number of neurons in the hidden layer is designed by the system, and whether clinical nurses suffer from sleep disturbance is taken as the output neuron. Finally, the structure of the neural network model is as follows: the input layer (14 neurons), the hidden layer (5 neurons), the output layer (2 neurons).The importance of each predictor is shown in Fig. 4.The normal importance ranking of each dependent variable is as follows: Burnout > Age > Depression > Anxiety > Chronic disease > Fatigue. According to the ANN model, the burnout was the predominant predictor of sleep disturbance (Fig. 4).

### Risk of sleep disturbance based on the nomogram scores

The sensitivity, specificity, positive predictive value, and negative predictive value when used in differentiating the presence from absence of sleep disturbance were 82.8%, 83.9%, 87.8%, and 78.0% in the training cohort, and 88.8%, 78.0%,84.5%, and 80.4% in the validation cohort, respectively (Table 4).

**Table 4:** Accuracy of the Prediction Score of the Nomogram for Estimating the Risk of Sleep Disturbance Presence

Variable	Value(95%CI)	
	Training Cohort	Validation Cohort
Area under ROC curve, concordance index	0.90(0.86–0.93)	0.89(0.83–0.95)
Cutoff value	0.53	0.55
Sensitivity, %	83.9(77.5,88.8)	88.8(79.2,94.4)
Specificity, %	83.1(75.0,89.0)	74.0(59.4,84.9)
Positive predictive value, %	87.8(81.7,92.1)	84.5(74.6,91.2)
Negative predictive value, %	78.0(69.8,84.6)	80.4(65.6,90.1)
Positive likelihood ratio	4.95	3.41
Negative likelihood ratio	0.19	0.15

## Discussion

In the current study ,nearly 60% of the clinical nurses were reported sleep disturbance. Comparing with recent studies, it can be concluded that the incidence of sleep disturbance in this study nurses was higher than that of Australian menopausal women 17] and general Asian population [18] Sleep disturbance is a medical disturbance of sleep patterns and the most significant cause of multiple psychological disturbance and somatic diseases [19] –21] The early identification of nurses at higher risk of sleep disturbances is critical to nursing career development. Gender, age, anxiety, night shift, etc. have identified multiple predictors to significantly correlated with sleep disturbance [22] –25] Nevertheless, our data showed that the gender, night shift had no relevance in the prediction of sleep disturbance. Besides, in this study, fatigue, chronic diseases, depression, burnout were independent risk factors for sleep disturbance in the multivariate logistic regression analysis. To our knowledge, this is the first nomogram and ANN model to predict the risk of sleep disturbance in clinical nurses.

### Age

As shown in the nomogram of this study, the older the age, the higher the sleep disturbance risk .Previous studies 26] have shown that symptoms of insomnia may be exacerbated with age. This may be because older nurses experience higher levels of work responsibilities and family needs, which increase chronic stress levels and make them particularly prone to sleep problems.Therefore, it is suggested that managers should communicate more with nursing members, understand their inner world, and appropriately help them alleviate the pressure on their work and life.Therefore, it is recommended that managers communicate with nursing members more to understand everyone’s inner world, and appropriately help everyone relieve the pressure in work and life.

### Chronic disease

The results of this study showed that the incidence of sleep disturbance in nurses with chronic diseases was 4.05 times higher than that in nurses without chronic diseases. With the increase of age, the health status of nurses become worse. As a large nursing workload is accompanied by a poor physical condition, it will reduce work efficiency, resulting in longer working hours, affecting the recovery of physical function, and ultimately affecting sleep quality [27] Therefore, nurses and managers must be aware of this, establish a complete physical examination system and working environment, and improve the quality of sleep for nurses.

### Fatigue

In clinical work, fatigue is an uncomfortable feeling of subjectively enduring tiredness, as well as a feeling of weakness when engaged in physical or mental activities, and these feelings are not relieved in the short term.Fatigue is the first influencing factor of sub-health [28] This study found that physical fatigue is an independent risk factor for sleep disturbance in nurses, which may be related to the mechanical and repetitive nursing operations of nurses and the disturbance of biological clock caused by long-term night shift. Scholars Zdanowicz T 29] also proved this point of view.For the problem of nurse fatigue, first of all, hospitals should improve the management mechanism to reduce the workload of nurses.According to the situation of different departments, reasonable scheduling, reduce the frequency of night shift and shift, so that nurses more regular life.

### Anxiety-depression

Anxiety depression is one of the common psychological barriers of nurse, is individual subjective experience negative emotional state, anxiety, depression and other negative emotions, the greater the impact on the quality of sleep, sleep rhythm disturbance, and poor sleep quality is to cause a decline in cognitive function, increase anxiety, depression, long-term repeatedly to form a vicious circle.The results of this study showed that the incidence of sleep disturbance in nurses with anxiety and depression was 3.44 and 3.73 times higher than those in normal mood.Slightly higher than the research results of foreign scholars Chueh K H [30] ] (OR = 1.07, OR = 1.16).It may be due to the fact that clinical nurses are faced with heavy nursing work and are in a state of mental and physical fatigue and stress for a long time, and the majority of clinical nurses are female, bearing the pressure of housework and children’s education at the same time, so psychological conflicts are easy to occur, resulting in psychological imbalance.Park C H 31] also confirmed this view.Therefore, hospitals should carry out extensive psychological training to help nurses master the correct emotional regulation methods, reasonably regulate their own emotions and improve their sleep quality, so as to effectively relieve work pressure and improve work efficiency.

### Burnout

Burnout is a series of symptoms related to the work environment and an individual’s slow response to chronic emotional and interpersonal pressure at work.Studies have found that job burnout not only affects the mental and physical of staff, and to cause a decline in its efficiency, absenteeism and turnover increased, the serious influence the stability of the nursing team and nursing career, and sleep disturbance is the individual in the most prone to the symptoms of job burnout situations, in this study, serious nurse job burnout rate of sleep disturbance is higher;Severe job burnout has the strongest predictive effect on sleep disturbance, which is similar to previous studies by Sayilan A A 32] .Therefore, the management personnel should reduce the emotional exhaustion of nurses, provide a platform for nurses to communicate with each other, and carry out targeted and professional psychological counseling for female nurses, and provide positive psychological support.Reduce the depersonalization of nurses, strengthen vocational training for nurses, constantly improve their professional quality and work skills, enhance their professional identity.

Based on the results of univariable and multivariable analyses, a predictive nomogram and ANN model were established to predict sleep disturbances. The training cohort data was used as internal validation and the validation cohort data was used as external validation to validate the accuracy of this nomogram. The C-statistic values provided sufficient predictive accuracy in both cohorts,and the calibration plots shows good consistency in the existence of sleep disturbance ,which indicated the high accuracy of the nomogram. The designed three layer feed forward ANN has good prediction performance with a sensitivity of 86.1%. Compared with traditional regression methods, ANN did not require a predefined mathematical relationship between the dependent and independent variables and could model any arbitrarily complicated nonlinear relationship. These advantages enable ANN to be a useful tool in solving the complex challenge of prediction.

#### Limitations

First of all, the study only explored the sleep disturbance of nurses in Jin zhou city.Limiting the universality of the findings and requiring validation in other cities.Secondly, the risk factors of sleep disturbance are complex, and there are many factors that need to be studied and predicted. This improves the accuracy of the prediction model to a certain extent.

## Conclusion

Age, chronic diseases, fatigue, anxiety,depression, and burnout were independent risk factors of sleep disturbance among clinical nurses. The nomogram and ANN model could effectively predict the risk of sleep disturbances.

## Data Availability

The data garnered during the current study and the final data set used for statistical analysis are available from the corresponding author on reasonable request.
